# ML2Motif—Reliable extraction of discriminative sequence motifs from learning machines

**DOI:** 10.1371/journal.pone.0174392

**Published:** 2017-03-27

**Authors:** Marina M. -C. Vidovic, Marius Kloft, Klaus-Robert Müller, Nico Görnitz

**Affiliations:** 1 Machine Learning Group, Technical University of Berlin, Berlin, Germany; 2 Department of Computer Science, Humboldt University of Berlin, Berlin, Germany; 3 Department of Brain and Cognitive Engineering, Korea University, Anam-dong, Seongbuk-gu, Seoul 136-713, Korea; Harbin Institute of Technology Shenzhen Graduate School, CHINA

## Abstract

High prediction accuracies are not the only objective to consider when solving problems using machine learning. Instead, particular scientific applications require some explanation of the learned prediction function. For computational biology, positional oligomer importance matrices (POIMs) have been successfully applied to explain the decision of support vector machines (SVMs) using weighted-degree (WD) kernels. To extract relevant biological motifs from POIMs, the motifPOIM method has been devised and showed promising results on real-world data. Our contribution in this paper is twofold: as an extension to POIMs, we propose gPOIM, a general measure of feature importance for arbitrary learning machines and feature sets (including, but not limited to, SVMs and CNNs) and devise a sampling strategy for efficient computation. As a second contribution, we derive a convex formulation of motifPOIMs that leads to more reliable motif extraction from gPOIMs. Empirical evaluations confirm the usefulness of our approach on artificially generated data as well as on real-world datasets.

## Introduction

Machine learning is emerging as crucial technology in science and industry [1–4]. The optimal choice of a learning method depends on the quality and quantity of the data, on the intrinsic noise characteristics and complexity underlying the data, and on the choice of an appropriate representation embracing the prior knowledge available. Lately, rather sophisticated, non-linear learning machines—such as kernel machines and deep neural networks—have become a gold standard in several application domains, including computational biology, image and speech recognition, and text mining. Unlike linear methods [5], these non-linear learning methods do not provide an explanation of the underlying prediction out of the box and are therefore generally considered as black boxes [6, 7].

In computational biology, positional oligomer importance matrices (POIMs) [8] have been successfully used to unveil the inner functions of kernel machines operating on DNA sequences. Originally aimed at categorical features only, POIMs have been later generalized to continuous features as the so-called feature importance ranking measure (FIRM [9]). Unfortunately, FIRM remains computational infeasible in most cases.

As visual inspecting POIMs can be tedious, [10, 11] proposed SVM2Motif, a probabilistic (non-convex) method to automatically extract the biological factors underlying the SVM’s prediction such as transcription factor binding sites or promoter elements –often called motifs. To extract motifs, the authors use a two-step approach where a POIM is extracted given a trained SVM classifier and compared against a corresponding motifPOIM that was generated by a set of proposal motifs. By varying these proposal motifs such that the distance between the classfier’s POIM and the generated motifPOIM is minimized, the desired motifs, underlying the classifier decisions, can be reconstructed.

As in SVM2Motif, the goal of this work is to extract biological meaningful motifs in a two-step approach. However, we extend the SVM2Motif techniques significantly to

- arbitrary learning machines (including SVMs and deep neural networks)
- convex generation of motifPOIMs
- new instance-based explanations.

Building upon POIMs and FIRM, we propose gPOIM, a measure of feature importance for arbitrary machines learning methods and feature sets. Unlike POIMs and FIRM, gPOIM enables us to not only assess model-based but also instance-based feature importances [6, 7, 12]. Experiments on artificially generated sequences and real-world data show the merits of our approach when compared to prevalent competitors. While our work is originally motivated by computational biology applications, the proposed measure of feature importance, gPOIM, is universally applicable. Furthermore, we derive a convex formulation of the formerly non-convex motifPOIM approach and show that this extension greatly improves accuracy and reliability of the found motifs. In reminiscence of the precursor, SVM2Motif, the combination of gPOIM with convex motifPOIM is named ML2Motif, as it generalizes the previous approach to arbitrary machine learning methods.

In extensive experiments on artificially generated data as well as on real world data sets we investigate the properties and show advantages of our method when compared to appropriate competitors.

## Preliminaries

In this section, we discuss the feature explanation techniques on which the proposed method builds upon: positional oligomer importance matrices and motifPOIMs, which are specifically designed for DNA sequences, and their generalization—the feature importance measure (FIRM), which can be used for arbitrary feature sets. An overview of the discussed methods and their respective definitions can be found in Table 1.

**Table 1:** Overview of methods.

Method	Symbol	Description	Ref.
POIM	*Q*_*k*_	A Positional Oligomer Importance Matrix of grade *k* (feature importances [8])	Def. 1
diffPOIM	Ω	Differential POIM summarizes importances across POIMs *Q*_*k*_ for multiple *k* [8]	Eq (4)
FIRM	*Q*_*f*_	Generalization of POIMs [9]	Def. 4
motifPOIM	*R*	Reconstruction of a POIM *Q*_*k*_ from motifs [10, 11]	Def. 2
This paper			
gPOIM	*S*_*ϕ*,*f*_(*t*)	Our proposed measure of feature importance	Def. 5
convex motifPOIM	*R*		Eq (9)

### Positional Oligomer Importance Matrices (POIMs)

Positional oligomer importance matrices (POIMs, [8]) are a methodology to visualize feature importances of kernel machines over a quadbit alphabet (i.e., {*A*, *C*, *G*, *T*}, as in the case of DNA sequences), taking into account inter-correlations between the features. The approach is based on the weighted degree string kernel [13–16], which compares two discrete sequences $x=\left(x_{1},\ldots ,x_{L}\right),\;x^{\prime }=\left(x_{1}^{\prime },\ldots ,x_{L}^{\prime }\right)\in A^{L}$ of length *L* over the alphabet A with |A|<∞, by counting the number of matches of their subsequences up to a given length *ℓ*_*max*_ $\kappa \left(x,x^{\prime }\right)=\sum_{\ell =1}^{\;\ell_{\max}}\sum_{j=1}^{L-\ell +1}\;1_{\left\{x{\left[j\right]}^{\ell }=x^{\prime }{\left[j\right]}^{\ell }\right\}}.$ Here *x*[*j*]^*ℓ*^ specifies the length-*ℓ* subsequence of *x* starting at position *j*. Thus, each entry in the explicit representation of a sequence Φ(*x*) in the decision function of the kernel SVM s(x)≔〈w,Φ(x)〉 corresponds to a valid positional subsequence *y* of length *ℓ* ∈ {1, …, *ℓ*_max_} starting at position *j* ∈ {1, …, *L* − *ℓ* + 1}.

An entry in this feature representation Φ(*x*) of the kernel SVM equals one if the positional oligomer *y* occurs in *x* at position *j* and zero otherwise. Any zero entries of Φ(*x*) do not contribute to the dot product, which is why we can write *s*(*x*) as a sum over the positional oligomer weights of *w*, which occur in x. Hence, we can rewrite the WD-kernel based scoring function as

$$s\left(x\right)=\sum_{\ell =1}^{\ell_{\max}}\;\sum_{j=1}^{L-\ell +1}\;w_{\left(x{\left[j\right]}^{\ell },j\right)}.$$

In the case of support vector machines (SVM) [17, 18], the bigger the absolute value |*s*(*x*)| of a sequence *x*, the more reliable is the decision sign(*s*(*x*)). For instance in the application of gene finding, *s*(*x*) would give large positive scores for sequences likely to contain genes and large negative scores for intergenetic sequences. Following up on this intuition, POIMs are formally defined as follows.

From now on, let *X* be a uniformly distributed random variable over the DNA alphabet Σ = {*A*, *C*, *G*, *T*} of length *L*.

**Definition 1** (POIM). *Given an SVM scoring function* *s* *based upon an WD-kernel of, at least, degree* *k* ≥ 1, *then for each possible* *k-mer* *y* *at position j we define the positional oligomer importance score as*

$$Q_{k,y,j}=E\left[s\left(X\right)|X{\left[j\right]}^{k}=y\right]-E\left[s\left(X\right)\right],$$

*which results, applied successively, in the positional oligomer importance matrix* *Q*_*k*_.

There are two reasons for subtracting E[s(X)] in the SVM POIM Def. 1. First, the expected value of the SVM scoring function can be considered as a baseline value, which is necessary for the interpretation of the conditioned expected value of the scoring function with respect to a single positional oligomer. The second and more important reason is the increased computation speed, since all non-overlapping positional oligomers do not have to be considered in the SVM POIM formula because their probability terms equal zero (cf. [10, 11]). Note that a glossery of the most used symbols is given in Table 2.

**Table 2:** Glossary of most important variables, functions, and symbols.

Symbol	Description
x∈X	Calligraphic upper case characters are input spaces for which the corresponding lower case characters are realizations
$1_{\left\{x=y\right\}}$	Indicator function (returns 1 if *x* = *y* else 0)
*s*(*x*)	Classifier scoring function (returns scalar score given an input instance *x*)
$\bar{s}\left(x|m_{k}\right)$	Reconstructed classifier scoring function given an input instance *x* and a motif *m*_*k*_
*L*	Length of a sequence
*X*[*j*]^*k*^	The subsequence within *X* starting at position *j* with length *k*
Φ(*x*)	Feature representation of the WD kernel
Σ	DNA alphabet {*A*, *C*, *G*, *T*}
PWM *r*	Positional weight matrix
PPM *m*_*k*_	A probabilistic positional motif (aka motif) consists of a PWM together and its starting position with variance

### Extracting motifs by mimicking POIMs

**Fig 1:**
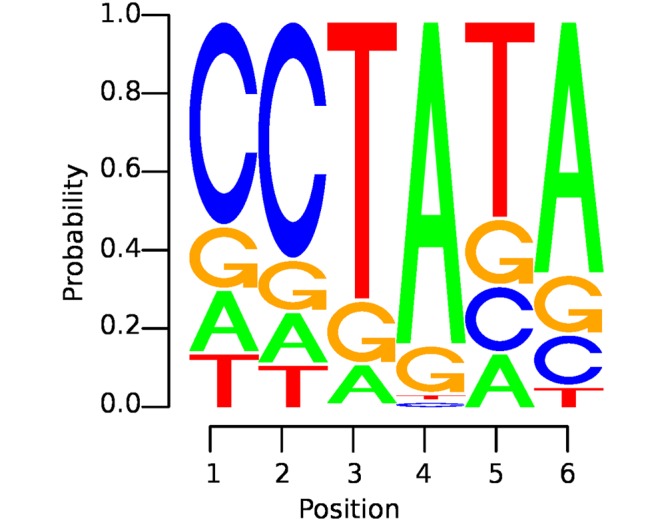
Example of a motif—i.e., an “interesting” subsequence of the DNA—illustrated as a positional weight matrix (PWM): the size of a letter indicates the probability of the occurrence of the corresponding nucleotide at a certain position in the motif. The likeliest nucleotides are arranged top down.

Extracting motifs means, extracting interesting subsequences of the DNA, such as transcription factors, start sites or promoter elements. In computational biology a motif is mostly indicated as positional weight matrix (PWM), which can be seen in Fig 1.

As useful as POIMs have proven to be, they can easily become too large for visual inspection. This results from the fact that their size grows exponentially with the length of the motif. This renders their computation feasible only for rather small motif sizes, typically *k* ≤ 12, but also manual inspection is hindered by the pure size of the matrix in order to determine relevant motifs. For example, while a POIM of order *k* = 5 contains 4^5^ ≈ 1,000 oligomers, slightly increasing the motif length to *k* = 10 leads to an unfeasible amount of 4^10^ ≈ 1,000,000 subsequences per position in the POIM [10].

**Fig 2:**
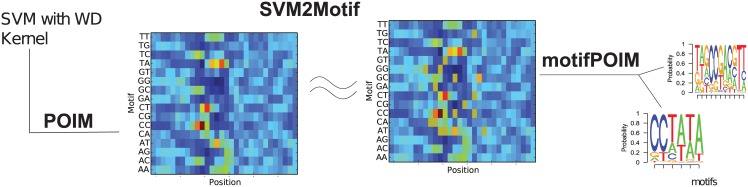
SVM2Motif. The SVM2Motif approach proceeds in two steps: First, feature importances are extracted from a SVM with weighted degree (WD) kernel resulting in a POIM (left). Second, a corresponding motifPOIM is constructed by a set of proposal motifs (right). Adjusting the proposal motifs such that the distance between gPOIM and the motifPOIM becomes minimal, gives the desired motifs.

Vidovic et al. [10, 11] present an elegant solution—motifPOIMs—to obtain short, relevant motifs even from an infeasible large POIM. The idea, shown in Fig 2, is to optimize a small set of proposal motifs to mimic the corresponding POIM using a probabilistic model (compare Fig 2).

We segment the method in its four substantial steps, which leads to a non-convex optimization problem:

1. *motif definition:* The proposal motifs are defined as probabilistic positional motifs(PPMs), which is a tuple *m*_*k*_ ≔ (*r*, *μ*, *σ*), where $r\in R^{|\Sigma |\times k}$ is a stochastic matrix (PWM, positional weight matrix) and codes for the motif and *μ*, σ∈R.
2. *motif weight function:* A PPM induces a probabilistic model. Given *μ* and *σ* as the starting position with its variance of the PPM, the Gaussian probability function for the starting position is
  $$P_{\left(z,i\right)}^{1}\left(m_{k}\right)≔\frac{1}{\sqrt{2\pi }\sigma }exp\;\left(-\frac{{\left(i-\mu \right)}^{2}}{2\sigma^{2}}\right).$$ (1)
  Furthermore, the probability of the motif sequence itself is given by the product of its PWM entries
  $$P_{\left(z,i\right)}^{2}\left(m_{k}\right)≔\prod_{\ell =1}^{k}\;r_{z_{\ell },\ell }.$$ (2)
  Combining *P*^1^ and *P*^2^, the probability for each oligomer at each position
  $$\begin{array}{c} v_{\left(z,i\right)}\left(m_{k}\right)≔P_{\left(z,i\right)}^{1}\left(m_{k}\right)\;P_{\left(z,i\right)}^{2}\left(m_{k}\right), \end{array}$$
  can be assembled and gives us a weight vector similar to the weight vector of the SVM.
3. *motif scoring function:* Thus, we are able to resemble the SVM scoring function as a motif scoring function:
  $$\bar{s}\left(x|m_{k}\right)≔\sum_{i=1}^{L-k+1}\;v_{\left(x{\left[i\right]}^{k},i\right)}\left(m_{k}\right).$$ (3)
4. *motifPOIM formula:* Consequently, we define in Def. 2 a motifPOIM *R* in analogy to the POIM *Q* (see Def. 1).

**Definition 2** (motifPOIM). *Given a motif scoring function* $\bar{s}$ *as defined in* Eq (3), *then for each possible* *k-mer* *y at position j we define a motifPOIM score as*

$$\begin{array}{c} R_{y,j}\left(m_{k}\right)≔E\left[\bar{s}\left(X|m_{k}\right)|X{\left[j\right]}^{k}=y\right]-E\left[\bar{s}\left(X|m_{k}\right)\right], \end{array}$$

*which results, applied successively, in the motifPOIM R*.

The main idea is to minimize the distance between the POIM and the motifPOIM, such that the PPM converges towards the true underling motif. To solve large problems in practice, Vidovic et al. [10, 11] split the long PPMs in shorter, overlapping SubPPMs (cf. appendix Def. S.1). However, this non-convex minimization problem leads to locally optimal solutions that can be enhanced by appropriate initialization, which may lead to more stability and reliability of the method. A greedy approach for initialization is given by differential POIMs [8]. Formally, the differential POIM Ω is defined as a *k* × *L* matrix Ω ≔ (Ω_*l*,*j*_) with entries

$$\begin{array}{c} \Omega_{l,j}≔\left\{\begin{array}{c} q_{\max}^{l,j}-\max\left\{q_{\max}^{l-1,j},q_{\max}^{l-1,j+1}\right\}\;\;\text{if}\;l\in \{2,\ldots ,L\}\\ 0\;\;\;\text{else}. \end{array}\right. \end{array}$$ (4)

where $q_{\max}^{l,j}≔\max_{y\in \Sigma^{l}}\;\left|Q_{l,y,j}\right|.$ Ω_*l*,*j*_ can be interpreted as overall importance of any oligomers of length *l* starting at position *j*. This can be used for initialization.

### Feature Importance Ranking Measure (FIRM)

Since POIMs are limited in applicability to DNA sequences, Zien et al. [9] introduced the feature importance ranking measure (FIRM), as a generalization of POIMs to arbitrary learning machines and features. FIRM consists of two steps. First, the score of a feature *f*(*X*) taking the value *t* is computed as follows.

**Definition 3** (Conditional expected score). *The conditional expected score of s for a feature f is the expected score* $q_{f}\;:\;R\to R$ *conditioned on the feature value t of the feature f:*

$$\begin{array}{c} q_{f}\left(t\right)=E\left[s\left(X\right)|f\left(X\right)=t\right]. \end{array}$$ (5)

The second step in FIRM establishes the variability of the conditional expected score as a measure for importance of the corresponding feature.

**Definition 4** (Feature importance ranking measure (FIRM)). *The feature importance Q*_*f*_ ∈ *R of the feature f is the standard deviation of the function* *q_f_:*

$$Q_{f}≔\sqrt{Var[q_{f}\left(f\left(X\right)\right]}.$$

FIRM has a variety of interesting properties. Above all, it is applicable for a wide range of machine learning methods and not even confined to features that have been used in the learning machine. This property has been tagged universal by [9]. In addition, POIMs are a special case for FIRM which, to some extend, is just an extension of POIMs to continuous features. Further, FIRM is robust to irrelevant transformations and objective when normalized properly. Albeit, it is sensitive to rescaling of the scoring function.

Interestingly, Zien et al. [9] discuss shortly the possibility of assessing all quantities empirical but let go of this idea “due to the limited amount of data“. The authors therefore present exact calculations for approximate features importances for certain settings (i.e. normally distributed data). In this work, we will argue for the simple solution of empirically assessing feature importances and show its advantages.

## Methodology

**Fig 3:**
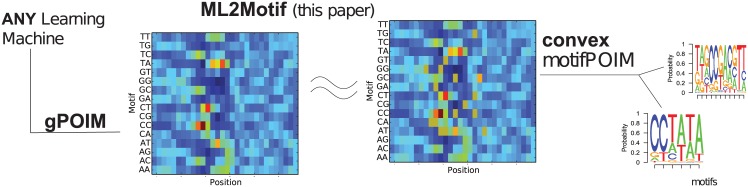
ML2Motif. Our approach proceeds in two steps: First, feature importances are extracted from a given learning machine resulting in a gPOIM (left). Second, a corresponding convex motifPOIM is constructed by a set of proposal motifs (right). Adjusting the proposal motifs such that the distance between gPOIM and the convex motifPOIM becomes minimal, gives us the desired motifs.

The contribution of this section is twofold: first, we devise a feature importance measure, which we call gPOIM, based on POIMs and its generalization (FIRM), and show that there is a simple way of assessing feature importances, enabling the extraction of importances from arbitrary learning machines. Second, we devise a convex version of the motifPOIM approach proposed by [10] and discuss its properties. Both methods combined form the basis of our motif extraction approach (ML2Motif, cf. Fig 3. ML2Motif follows the same principles as SVM2Motif (cf. Fig 2).

### gPOIM—Generalizing POIMs

In this section, we devise a new method —gPOIM— for extracting feature importances from arbitrary learning machines. It builds on the concepts of FIRM and POIMs, addressing their shortcomings.

POIMs and FIRM are notoriously difficult to implement and/or evaluate in practice (to achieve reasonable runtime performance). POIMs are restricted to specific learning machines (kernel machines using a WD kernel over DNA sequences). The feature importance ranking measure (FIRM, [9]), on the other hand, is a general and theoretically appealing concept, which generalizes POIMs. However, computation is in general intractable [9] except for a few special cases such as linear Gaussian models or WD-kernels over (discrete) sequences.

In contrast, gPOIM can be easily computed for any learning machine, including deep learning, and any feature set ([19, 20]). Furthermore, we propose a fast and simple sampling-based approach for gPOIM, which greatly simplifies implementation and evaluation.

**Definition 5** (gPOIM). *Let X be a uniformly distributed random variable on a space* X. *We define the gPOIM measure as follows. Furthermore, let* s:X→Y *be a prediction function (output by an arbitrary learning machine), and let* f:X→R *be a real-valued feature. Let* ϕ:X→F *be a function (“explanation mode”), where F is an arbitrary space. Then we define gPOIM as:*

$$\begin{array}{c} S_{\phi ,f}^{*}\left(t\right)≔E\left[s\left(X\right)\phi \left(X\right)|f\left(X\right)=t\right]. \end{array}$$ (6)

**Fig 4:**
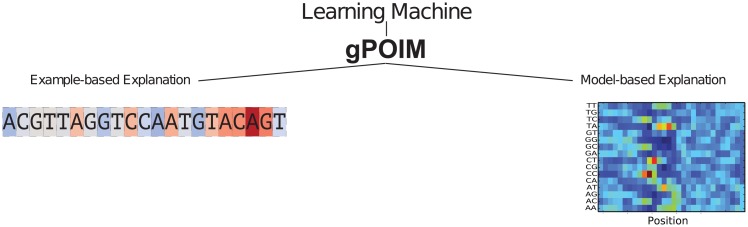
gPOIM Explanation modes. We consider two possible settings for measuring feature importances: (left) instance-based importance measure (e.g. Why is this specific example classified as positive using my trained classifier?); (right) model-based importance measure (e.g. Which features are generally important for my trained classifier?).

In many ways, gPOIM reflects the conditional expected score as defined for POIMs and FIRM (cf. Def. 1 and Def. 3). However, there are certain extensions made possible by the “explanation mode” of the above definition, which gives us some more degrees of freedom as illustrated in Fig 4 and as confirmed in the empirical section of this paper. Now, we explain the “explanation mode” in terms of a model-based and an instance-based procedure exemplary for sequence data.

#### Model-based gPOIM

Here, the task is to globally assess what features a given (trained) learning machine regards as most significant—independent of the examples given and hence, we neglect *f*(*X*) = *t*. In the case of sequence data, where sequences of length *L* over the alphabet Σ = {*A*, *C*, *G*, *T*} are given, an importance map for all *k*-mers over all positions is gained by using the explanation mode *ϕ*: Σ^*L*^ → Σ^*k*×{1,…,*L*−*k*+1}^, where each sequence is mapped to a sparse PWM, in which entries only indicate presence or absence of positional *k*-mers:

$$\begin{array}{c} S_{\phi }^{*}≔E\left[s\left(X\right)\phi \left(X\right)\right]. \end{array}$$

#### Instance-based gPOIM

Given a specific example, the task at hand is to assess why this example has been assigned this specific classifier score (or class) prediction. In the case of sequence data we compute the feature importance of any positional *k*-mer in a given sequence *g* ∈ Σ^*L*^ by *f*(*X*) = *X*[*i*]^*k*^, with *t* = *g*[*i*]^*k*^:

$$\begin{array}{c} S_{f}^{*}\left(t\right)≔E\left[s\left(X\right)|f\left(X\right)=t\right]. \end{array}$$

#### Computation

In order to make this approach practically suitable, we resort to sampling as an inference method. To this end, let Z⊂X be a subset of X containing *n* = |*Z*| samples, Eq (6) can be approximated by

$$\begin{array}{c} S_{\phi ,f}\left(t\right)≔E_{X=Z}\left[s\left(X\right)\phi \left(X\right)|f\left(X\right)=t\right]=\frac{1}{|Z_{\left\{f(z)=t\right\}}|}\;\sum_{z\in Z}\;s\left(z\right)\phi \left(z\right)1_{\left\{f(z)=t\right\}}, \end{array}$$ (7)

where *Z*_{*f*(*z*) = *t*}_ ⊆ *Z* contains only elements for which *f*(*z*) = *t* holds. It holds true that if the number of samples |*Z*| → ∞ then $S_{\phi ,f}\to S_{\phi ,f}^{*}$.

To simplify notation and to resemble POIMs for subsequent analysis, we re-index gPOIM for model-based explanation as follows

$$\begin{array}{ccc} S_{k,y,j}≔\left|Z_{\left\{X{\left[j\right]}^{k}\neq y\right\}}\right|\cdot S_{\phi_{k,y,j}} & = & |Z_{\left\{X{\left[j\right]}^{k}\neq y\right\}}|\cdot E_{X=Z}\left[s\left(X\right)\phi_{k,y,i}\left(X\right)\right]\\ & = & |Z_{\left\{X{\left[j\right]}^{k}\neq y\right\}}|\cdot E_{X=Z}\left[s\left(X\right)1_{\left\{X{\left[j\right]}^{k}=y\right\}}\right]\\ & = & E_{X=Z}\left[s\left(X\right)|X{\left[j\right]}^{k}=y\right], \end{array}$$ (8)

which gives us the unnormalized POIM formulation of Def. 1.

### Convex motifPOIMs—Fast and reliable motifPOIMs

In genomic discrimination tasks, underlying motifs are of prime importance, as they resemble the biological objects of interest, e.g., splice sites, gene starts, or transcription factors. Unfortunately, similar to POIMs (see Def. 1), gPOIMs grow exponentially with the size of the motifs, which renders manual inspection impossible even for small motif sizes. In order to extract the relevant motifs automatically, the motifPOIM approach was proposed and showed promising results. However, devised as a highly non-convex optimization problem, motifPOIM optimization generally leads to a sub-optimal local minimum and therefore may be less stable and reliable. Furthermore, motifPOIMs mimic SVMs, which reduces generality of the approach.

In the following, we improve motifPOIM to achieve a simpler, faster, more general, and—above all—convex approach. Therefore, relevant motifs are defined as PWM *m*_*k*_ = (*r*, *μ*), where $r\in R^{4\times k}$ induce a probabilistic model

$$v_{z}\left(m_{k}\right)=\prod_{\ell =1}^{k}\;r_{z_{\ell },\ell },$$

which calculates the probability for the representation of *k*-mer *z* solely as a product of its PWM entries (hence, omitting Eq (1) due to σ* < < 1 in most applications and *P*^1^ ∼ 1 for *i* = *μ* and 0 otherwise). With a given motif environment $U\left(m_{k}\right)≔U\left(\mu \right)≔\left[\mu ,\ldots ,\mu +k-1\right]$ and SubPPMs ${\tilde{m}}_{i-\mu }\left(m_{k},\tilde{k}\right)$ (see appendix Def. S.1) for i∈U(μ), we define the motifPOIM score as:

$$R_{\left(z,i\right)}\left(m_{k}\right)≔1_{\left\{i\in U\left(m_{k}\right)\right\}}v_{z}\left({\tilde{m}}_{i-\mu }\left(m_{k},\tilde{k}\right)\right).$$ (9)

Finally, this leads to the following objective function:

$$\begin{array}{ccc} f({\left(m_{k,t}\right)}_{t=1,\ldots ,T_{k},k\in K}) & = & \frac{1}{2}\;\sum_{k\in K}\;\sum_{y\in \Sigma^{\tilde{k}}}\;\sum_{j=1}^{L-\tilde{k}+1}\;(\sum_{t=1}^{T_{k}}R_{y,j}\left(m_{k,t}\right)-S_{\tilde{k},y,j}{)}^{2} \end{array}$$ (10)

Note, that in Eq (1) the expected value of *s*(*X*) was subtracted, which was done to reduce the computational cost [21]. However, in Eq (6) we do not have to subtract the expected value of *s*(*X*) since gPOIM has no time consuming iteration over the whole set of possibilities. That this leads to no reduction for the optimization is stated in the following theorem. Note, from now, to improve the readability, we restrict the extraction to one motif of fix length *k*, only. The theorems and proofs for the case of multiple motifs can be found in the Supplementary.

**Theorem 1.** *Suppose that the objective function* *f*(*r*; *μ*) *of*

$$\begin{array}{ccc} & \min_{r} & f\left(r;\mu \right)=\frac{1}{2}\;\sum_{y\in \Sigma^{\tilde{k}}}\;\sum_{j=1}^{L-\tilde{k}+1}\;{\left(R_{y,j}\left(m_{k,t}\right)-S_{\tilde{k},y,j}+c\right)}^{2}\\ & s.t. & 0\leq r_{o,s}\leq 1\;o=1,\ldots ,4,s=1,\ldots ,k,\\ & & \sum_{o}\;r_{o,s}=1\;s=1,\ldots ,k. \end{array}$$ (11)

*is convex and let* $r_{c}^{*}$ *be the optimal solution, then* $\forall c^{\prime }\in R\;r_{c^{\prime }}^{*}=r_{c}^{*}$.

*Proof*. Let $r_{c}^{*}$ be the optimal solution of the objective function *f* Eq (11) with the inequality constraints *h*_*o*,*s*,1_ = −*r*_*o*,*s*_ and *h*_*o*,*s*,2_ = *r*_*o*,*s*_ − 1, *o* = 1, …, 4, *s* = 1, …, *k*, *i* = 1, 2 and the equality constraints *g*_*s*_ = ∑_*o*_ *r*_*o*,*s*_ − 1, *s* = 1, …, *k*, and let *η* and *ξ* be the Lagrangian multipliers, then the Lagrangian function is as follows

$$\begin{array}{c} L\left(r_{c}^{*},\eta ,\xi \right)=f\left(r_{c}^{*};\mu \right)+\sum_{o=1}^{4}\;\sum_{s=1}^{k}\;\eta_{o,s,1}h_{o,s,1}+\sum_{o=1}^{4}\;\sum_{s=1}^{k}\;\eta_{o,s,2}h_{o,s,2}+\sum_{s=1}^{k}\;\xi_{s}g_{s}. \end{array}$$

The Karush-Kuhn-Tucker(KKT) conditions are satisfied for $r_{c}^{*}$: For the dual feasibility conditions (*η* ≥ 0) and, since *r* is a stochastic matrix, the primal and the complementary slackness conditions (*g*_*s*_ = 0, *s* = 1, …, *k*, *h*_*o*,*s*,*i*_ ≤ 0, *o* = 1, …, 4, *s* = 1, …, *k*, *i* = 1, 2, and *η*_*o*,*s*,*i*_ *h*_*o*,*s*,*i*_ = 0, *o* = 1, …, 4, *s* = 1, …, *k*, *i* = 1, 2) are trivially fulfilled, which leaves us to show that the stationarity condition

$$\nabla f\left(r_{c}^{*};\mu \right)+\sum_{i=1}^{2}\;\sum_{o}\;\sum_{s=1}^{k}\;\eta_{o,s,i}\nabla h_{o,s,i}+\sum_{s}^{k}\;\xi_{s}\nabla g_{s}=0$$

is satisfied. Therefore we insert the derivations and reorganize for the Lagrange multipliers *ξ*, which leads to

$$\begin{array}{c} \xi_{s}=-\sum_{y}\underset{j}{\sum }\;1_{\left\{i\in U\left(\mu \right)\right\}}\;\left(\prod_{l=1}^{\tilde{k}}r_{y_{l},j+l}^{*}\prod_{\begin{array}{l} l=1\\ l\neq t \end{array}}^{\tilde{k}}r_{y_{l},j+l}^{*}-\left(S_{\tilde{k},y,j+\mu }-c\right)\prod_{\begin{array}{l} l=1\\ l\neq t \end{array}}^{\tilde{k}}\;r_{y_{l},j+l}^{*}\right)\;+\eta_{o,s,1}+\eta_{o,s,2}.\; \end{array}$$

With ξ∈R it holds, that for any $c^{\prime }\in R\;r_{c^{\prime }}^{*}=r_{c}^{*}$. The fact that *f* is convex, *h* is convex, and *g* is affine implies that the KKT conditions are sufficient for optimality and thus concludes the proof.

The proof leaves us to show the convexity of function *f* in Eq 11.

**Theorem 2** (Convexity). *Let D be a convex set, m_k_* ∈ *D a probabilistic motif, S a gPOIM, such that* $S_{\tilde{k},y,j}\in R$ *for* $y\in \Sigma^{\tilde{k}}$ *and* $j=1,\ldots ,L-\tilde{k}+1$, *μ* ∈ [1, *L* − *k* + 1], c∈R *and S*_⌊_ *the element wise minimum of S then, if* $c\geq 1_{\left\{S_{\lfloor }<0\right\}}S_{\lfloor }+1_{\left\{\min(S)<1\right\}}$ *it holds that*

$$\begin{array}{ccc} f(m_{k}) & = & \frac{1}{2}\;\sum_{y\in \Sigma^{\tilde{k}}}\;\sum_{j=1}^{L-\tilde{k}+1}\;{\left(R_{y,j}\left(m_{k}\right)-\left(S_{\tilde{k},y,j}+c\right)\right)}^{2} \end{array}$$ (12)

*is convex*.

*Proof*. We have to proof the following inequality

$$\begin{array}{ccc} ||R\left(\Phi r+\left(1-\Phi \right)s;\mu \right)-\left(S+c^{\prime }\right){\left|\right|}_{2}^{2} & \leq & \Phi ||R\left(r;\mu \right)-\left(S+c^{\prime }\right){\left|\right|}_{2}^{2}\;+\;\left(1-\Phi \right)||R\left(s;\mu \right)-\left(S+c^{\prime }\right){\left|\right|}_{2}^{2} \end{array}$$

to show convexity of *f*(*m*_*k*_), which is, for the case $j\notin 1_{\left\{i\in U(\mu )\right\}}$, trivially fulfilled for $c^{\prime }\in R$. This, due to the fact, that a sum of convex functions is convex, leaves us with showing the following inequality

$$\begin{array}{c} {\left(\Phi a+\left(1-\Phi \right)b-\left(S_{\tilde{k},y,j}+c^{\prime }\right)\right)}^{2}\leq \Phi {\left(a-(S_{\tilde{k},y,j}+c^{\prime })\right)}^{2}+\left(1-\Phi \right){\left(b-(S_{\tilde{k},y,j}+c^{\prime })\right)}^{2}, \end{array}$$ (13)

where we replaced the PWM products $\prod_{l=j}^{k+j}\;r_{y_{l},l}$ and $\prod_{l=j}^{k+j}\;s_{y_{l},l}$ by *a* and *b* for more transparency. After resolving and transforming Eq (13) shortens to

$$\Phi^{2}a^{2}+2\Phi ab-2\Phi^{2}ab\leq \Phi a^{2}+2\Phi {\left(S_{\tilde{k},y,j}+c^{\prime }\right)}^{2}.$$

Since −2Φ^2^ *ab* ≤ 0 and Φ^2^ *a*^2^ ≤ Φ*a*^2^, the equation reduces to

$$ab\leq {\left(S_{\tilde{k},y,j}+c^{\prime }\right)}^{2}.$$

The fact that the maximum of *ab* is 1, concludes the proof for *c* ≥ *c*′ with $c^{\prime }=1_{\left\{\min(S)<0\right\}}S_{\lfloor }+1_{\left\{\min(S)<1\right\}}$.

## Empirical evaluation

The empirical evaluation has three parts: First, we investigate and discuss the properties of our proposed methods gPOIM and the corresponding convex motif extraction method when compared to their predecessors on artificially generated data. In the second part, we apply ML2Motif (=gPOIM and convex motifPOIM) to find driving motifs in real-world human splice-site data where ground truth motifs are known. Here, we compare motif reconstruction accuracies against state-of-the-art competitors under various experimental settings. Finally, we perform an analysis of the publicly available enhancer dataset and try to find and verify the driving motifs in a real-world setting where no ground truth motifs are given.

Since we focus on computational biology settings and specifically on the important task of motif finding in DNA sequences, we measure the accuracy of predicted motifs in terms of motif reconstruction quality [22]

$$\text{MRQ}=\sum_{p=1}^{k}\;\left[\frac{1}{k}-\frac{1}{2k}\;\sum_{c\in \{A,C,G,T\}}\;{\left(t_{cp}-r_{cp}\right)}^{2}\right]\;,$$

where the ground truth sequence motif is denoted *t* and the corresponding predicted motif *r*. As in [10, 11], we use differential POIMs (cf. Eq 4) to estimate position and length of motifs.

### Controlled experiments

In this section, we assess and discuss the properties of both, gPOIM and convex motifPOIMs. We start by showing the benefits of instance-based explanations, a new mode of explanation which was made possible by gPOIM. Further, we continue to discuss gPOIM in the traditional model-based explanation mode and compare solutions against its predecessor (POIM) in a variety of experiments. Finally, we show that convex motifPOIMs are able to extract complex motifs and unleash the full potential of our ML2Motif (=gPOIM and convex motifPOIM) by application to convolutional neural networks.

#### Instance-based explanation of DNA sequences

**Fig 5:**
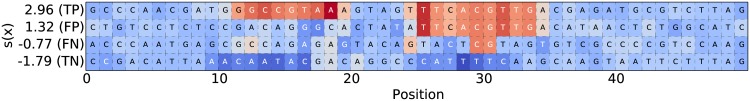
Instance-based explanation. Instance-based explanation of the SVM decision for samples coming from the true positive (TP), false positive (FP), false negative (FN) and true negative (TN) test set, respectively. The highlighted nucleotids are informative for the individual SVM decisions (scoring function on the y-axis).

For the instance-based experiment, we used 10,000 randomly generated sequences, with two motifs, (“GGCCGTAAA”,pos = 11) and (“TTTCACGTTGA”, pos = 24) placed in one quarter for training an SVM with an WD kernel. The SVM achieves an accuracy of 98,63%. In the following we explain the classifier decision for individual test sequences by subsequently explaining one example from the sets of the true positives, false positives, false negatives and true negatives test samples. The number of random samples for the gPOIM computation (Eq 7) comprises 10,000 samples. From the results, shown in Fig 5, we observe that the nucleotides building the two patterns have a strong discriminative evidence. If the discriminative patterns are too noisy, the sequences are classified to the negative class, which, in case of the false negative (FN) example leads to mis-classifications. Elsewise, if only one of the two patterns was inserted, the classifier gives high evidence to the single pattern, which also leads to mis-classification.

#### Model-based explanation of DNA sequences

We generated randomly 10,000 sequences of length 30, where positive examples carry the motif CCTATA at position 11. As classifiers we employ support vector machines with weighted degree kernel (degree = 8) and convolutional neural networks with following architecture: a 2D convolution layer with 10 tanh-filters of size 8x4, a max-pool layer of size = 2, a dense-layer with 100 ReLUs and a 1 dense layer with 2 softmax units.

**Fig 6:**
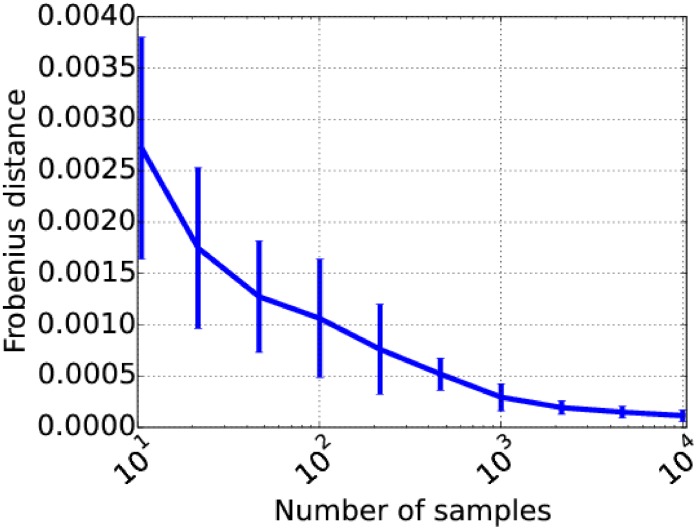
Reconstruction accuracy of gPOIM. Visualization of the reconstruction accuracy of gPOIM when compared to POIM for an increasing number of samples, measured by Frobenius distance.

To show that gPOIMs converges fast towards POIMs, we measured the Frobenius distance between gPOIMs and POIMs for an increasing number of samples used to build our gPOIM. In average, 1000 samples are enough to cross a 10^−3^ error bound. The experiment was repeated 25 times and mean as well as standard deviations are reported in Fig 6.

**Fig 7:**
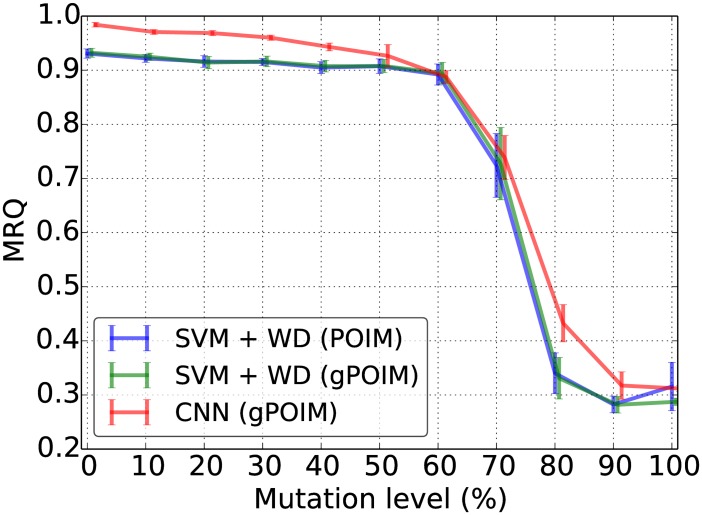
Accuracy comparison. The MRQ of SVM+POIM+convex motifPOIM, SVM+gPOIM+convex motifPOIM and CNN+gPOIM+convex motifPOIM for various levels of mutation in the data set.

Subsequently, as shown in Fig 7, we investigate the stability and accuracy of gPOIMs (using 1000 samples, green line) under noise when compared against the computed POIM (blue line) as implemented in the Shogun machine learning toolbox [23] (only available for linear SVMs with weighted-degree kernel though).

Noise was induced by mutating each of the nucleotides of the underlying motif with some probability (x-axis). As can be seen, there is virtually no difference between both methods for the same classifier using convex motifPOIM. Hence, we established that gPOIMs are a valid replacement for POIMs. To fully take advantage of the gPOIMs approach, we are able to use more complex classifiers, e.g. CNNs (red line) which shows superior behavior. The drop after a noise level of 60% can be explained as follows. At a noise level of 66.6% all motifs have equal probability, which is why above that level, other motifs become more likely than the inserted motif. Hence, due to the considerable rarity of the motif at 66.6% the classifiers ability drops significantly.

#### Motif extraction by mimicking gPOIMs

**Fig 8:**
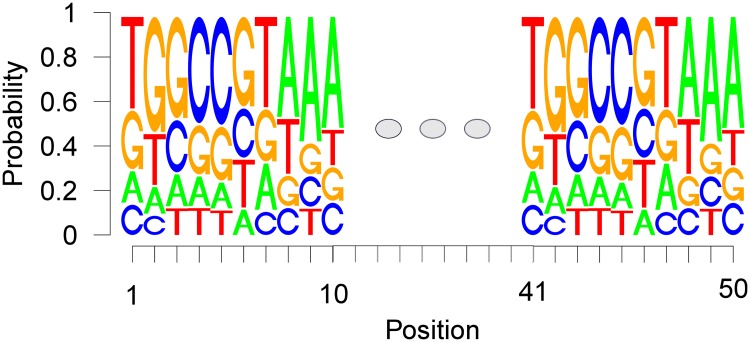
Long motif extraction. An induced ground truth motif of length 50 with the recurrent pattern TGGCCGTAAA is reconstructed concisely from noisy data.

To show whether or not we are able to find long motifs with our proposed method, we draw 10.000 uniformly distributed toy DNA sequences of length 100, where we insert a motif of length 50 at position 10 in 25% of the data. The motif pattern was of the form TGGCCGTAAA, which was inserted five times in a row. From the results, shown in Fig 8, we can observe that the real motif was found correctly.

**Fig 9:**
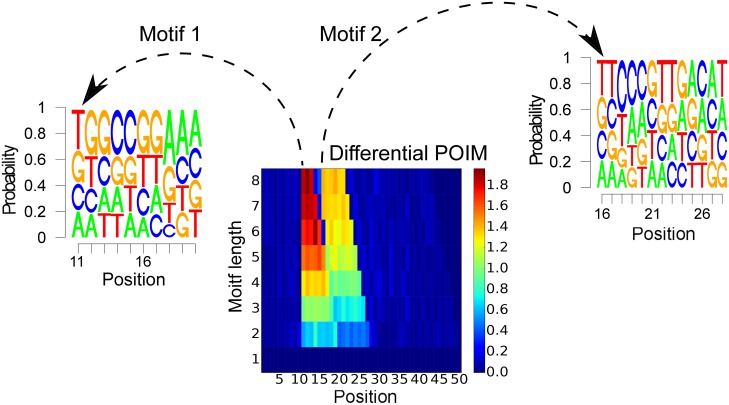
Overlapping motifs extraction. Based on the differential POIM (center), the estimated starting positions of the motifs are 11 and 16. Arrows point to the extracted motifs with highest scoring sequences coinciding with the induced ground truth motifs. Motifs are overlapping from positions 16 to 21.

In the following, we show that our method is capable of handling the difficulty of finding motifs that overlap each other, which means, motifs are sharing at least one position. For the experiment, we generate 1000 random sequences, where we placed the motifs (“TGGCCGGAAA”, 11) and (“TTCCCGTTGACAT”, 16) in 125 sequences, respectively. The results are shown in Fig 9, where we observe that the highest probability is given to the truly underlying motifs. The starting positions of the motifs were extracted from the differential POIM, which is shown in the center of Fig 9.

**Fig 10:**
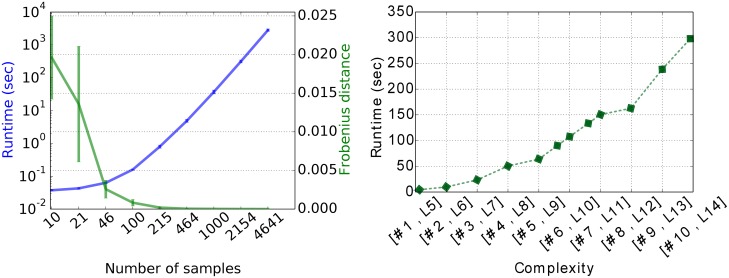
Runtimes. Left: Runtime (in seconds) for increasing number of samples (blue) and corresponding Frobenius distance of two consecutive results (saturation curve, green). Right: Runtime (in seconds) for increasing complexity (number # and length L of motifs).

Furthermore, we investigate the runtime behavior of the presented method. We aim to show two key results. First, the algorithm should produce an adequate gPOIM, which can be measured by the Frobenius distance to the true POIM, in a reasonable time. We can observe from the left side of Fig 10 that the runtime increases when at the same time the Frobenius norm between gPOIM and the true POIM decreases. After already 25 sec. we observe an accuracy of 10^−4^. Second, the optimization procedure should be computable in a reasonable time, also for complex motif finding problems. Therefore we measured the runtime for increasing complexity, i.e. increasing number of motif and motifs length. The results are shown on the right side of Fig 10. The runtime increases almost linear with the complexity of the program. Both experiments together show that our method is computable in reasonable times.

### Motif extraction from human splice-site data

We evaluate our proposed methods (gPOIM and convex motifPOIM) on a human splice site data set (sequences of length = 141 nucleotides, 1 true motif at position = 46 with length = 20, using a ratio = 0.25 positives/(positives+negatives)), which can be downloaded from http://www.fml.tuebingen.mpg.de/raetsch/projects/lsmkl. We used POIM and motifPOIM as baseline methods and MEME [24] as the state-of-the-art competitor. For verifying our results we employ the splice site motifs given by the JASPAR database [25] (Available from http://jaspar.genereg.net) as ground truth.

**Fig 11:**
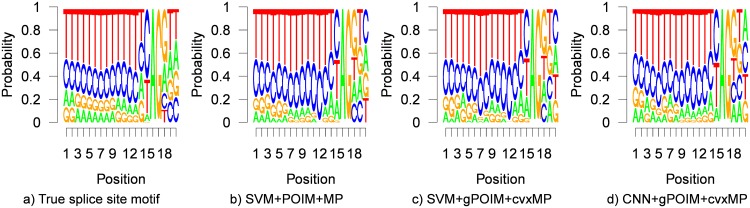
Inferred motifs (human splice sites experiment). a) true motif given by the JASPAR database, b) predicted motif from SVM+POIM+motifPOIM (MRQ = 97.05), c) SVM+gPOIM+convex motifPOIM (MRQ = 99.54), and d) CNN+gPOIM+convex motifPOIM (MRQ = 99.56).

The results in Table 3 show the mean and standard deviations of the MRQ accuracies for various numbers of training examples and 10 repetitions of each experiment. For all experiments, besides for the MEME motif finder, we employ a weighted degree kernel for the SVM with degree = 8 setting hyper-parameters according to [10, 11]. Using POIMs as implemented in the Shogun machine learning toolbox [23] (only available for SVMs with weighted degree kernel), we test the (non-convex) motifPOIM method against our convex motifPOIM. The resulting lower standard deviations indicate that our convex motifPOIM approach is more reliable than its non-convex predecessor. Furthermore, we gain almost 2% MRQ due to its inherently stable behavior. Next, we compare the results when using our gPOIM (1000 samples) instead of the Shogun implemented POIM. Here, we observe that the results are indistinguishable and thus, empirically justifying our sampling approach on non-trivial real-world data. Having established gPOIM as a valid approach for replacing POIMs, we proceed by taking advantage of its full potential and apply convolutional neural networks with following architecture: a 2D convolution layer with 10 tanh-filters of size 8x4, a max-pool layer of size = 2, a dense-layer with 100 ReLUs and a 1 dense layer with 2 softmax units. The architecture is similar to the one used in [26] and gives similar, almost perfect, results as can also be seen in Fig 11. As can be seen in Table 3, (g)POIM-based approaches outperform the MEME motif finder, which did not converge in reasonable time (>20h) for 30,000 sequences. Also, for less than 6,000 samples, MEME seems rather unstable as indicated by the high standard deviations.

**Table 3:** Results (human splice site experiment).

		POIM		gPOIM	
#	MEME	SVM+MP	SVM+cMP	SVM+cMP	CNN+cMP
300	89.31±5.27	97.79±0.37	98.77±0.17	98.97±0.24	98.94±0.26
600	90.02±2.86	97.91±0.24	99.16±0.14	99.18±0.14	99.17±0.14
1,200	92.66 ±4.99	97.49±0.13	99.36±0.10	99.25±0.03	99.32±0.13
2,400	93.18 ±4.18	97.61±0.24	99.37±0.07	99.38±0.06	99.37±0.05
6,000	94.70 ± 0.17	97.91±0.31	99.42±0.14	99.45±0.06	99.44±0.06
30,000	-	97.05±0.09	99.39±0.08	99.54±0.02	99.56±0.02

MRQ values and standard deviations for the human splice data set comparing our convex motifPOIMs and gPOIM against baseline methods (POIM and motifPOIM respectively) and state-of-the-art competitor MEME. The SVM was trained using weighted degree kernels. Due to lack of space, MP is the abbreviation for motifPOIM and cMP for convex motifPOIM.

**Fig 12:**
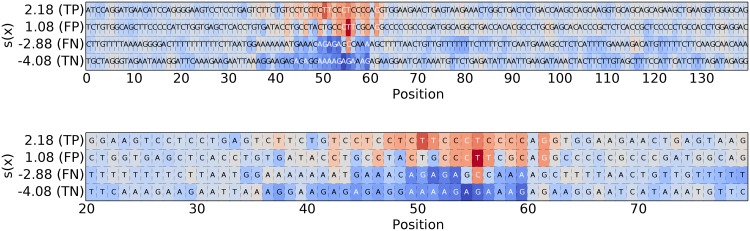
Instance-based explanation (human splice-site experiment). Position-wise importances for four specific sequences from the human splice site data set: (a) a true positive with high positive score *s*(*x*), (b) a false positive with low positive score *s*(*x*), (c) a false negative with low negative score *s*(*x*), and (d) a true negative with high negative score *s*(*x*). Upper figure shows whole sequences, lower figure is a zoomed-in version for better readability.

The use of gPOIMs enables us to not only extract motifs based on the trained model, instead, we are able to explain classifier decisions for specific sequences. Fig 12 shows the position-wise importances for 4 different sequences (true positive, false positive, false negative, and true negative) for the full 141 nucleotide sequence and a zoomed-in version. As can be seen, most important (dark blue and red) regions are around the true underlying sequence motif site with red for higher scores *s*(*x*) and blue for lower/negative classifier scores *s*(*x*).

### Exploratory analysis of enhancers and their strength

For most applications, there will be no ground truth motifs available in advance. To give an example on how to apply and verify ML2Motif in this real-world situation, we chose to test our method on an enhancer dataset supplied by [27], which can be downloaded at http://bioinformatics.hitsz.edu.cn/iEnhancer-2L/data. The data set comprises 742 weak enhancers, 742 strong enhancers and 1484 non-enhancers, each with a sequence length of 200 respectively.

Following [27], we build a two-layer classification framework, where the first layer decides whether or not the given sample is an enhancer. In case of positive prediction, the second layer will predict the enhancers strength. For both layers we trained an SVM (*C* = 1) with an WD kernel (kernel degree *k* = 8), where the first layer was trained on non-, strong, and weak enhancers and the second layer on strong (+1 class) and weak (-1 class) enhancers only. A 5-fold cross validation was applied to test prediction accuracy (classification accuracy). Here, we report a 95% accuracy for the first layer and 90% for the second layer. Both methods exceed the given baseline method (iEnhancer-2L, 76.89% and 61.93%, respectively) by a comfortable margin, which we claim on the richer feature representation (i.e. weighted degree kernel vs. RBF kernel).

**Fig 13:**
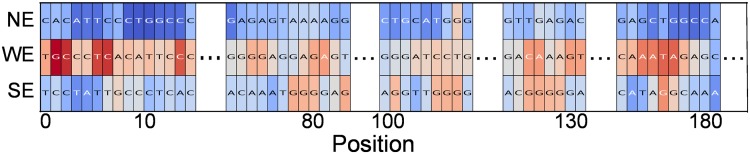
Instance-based explanation (enhancer experiment, layer 1). Instance-based explanation of one sample of each type, strong enhancers (SE), weak enhancers (WE), and non-enhancers (NE). Due to length of the sequences, only relevant parts of the instanced-based explanation are shown. We can observe that there are multiple relevant motifs, which also depend on the enhancer type (WE or SE).

**Fig 14:**
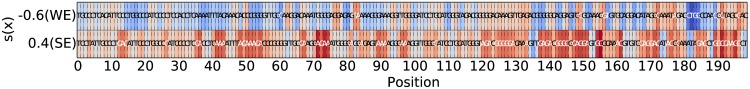
Instance-based explanation (enhancer experiment, layer 2). Position-wise importances are shown for a strong enhancers (SE) and a weak enhancers (WE) sequence.

If we apply ML2Motif to the SVM solution, we can have a first glimpse at the problem by using the instance-based explanation mode for a set of randomly chosen sequences of differing classes (cf. Figs 13 and 14). We observe that importances spread over the whole sequence length. This could be a hint that either multiple motifs spread over the whole sequence or that motifs are not located (=they can change position). Moreover, the importances include almost exclusively Guanine-sequences for the enhancer class. Hence, extracted motifs should contain strong Guanine components.

**Fig 15:**
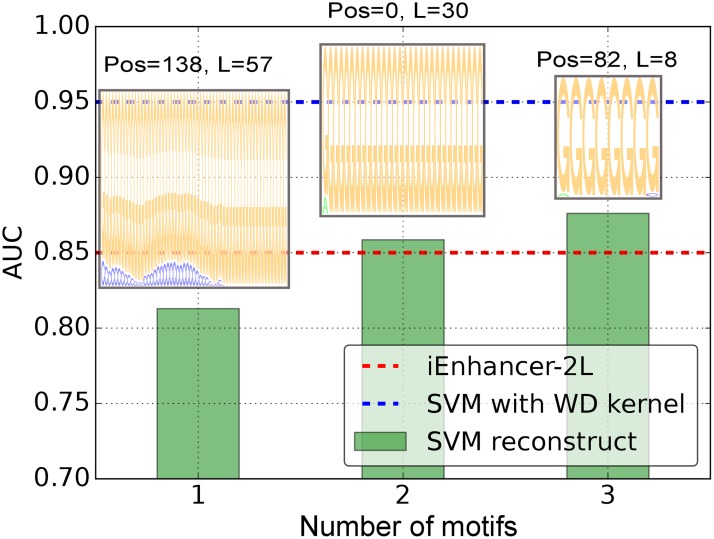
Classification accuracy (enhancer experiment, layer 1). An SVM with WD kernel (blue dashed line) was trained to discriminate between enhancers and non-enhancers and archives superior AUC of 95% when compared to the baseline method iEnhancer-2L (red dashed line, AUC of 85%). Subsequently, ML2Motif was applied to extract *n* ∈ {1, 2, 3} most significant motifs (x-axis) from the SVM classifier. To test their respective relevance, test sequences were ranked according to the extracted motifs. Results show (green bars with corresponding motif plotted on top) that two motifs suffice to surpass the baseline method.

Using again diffPOIMs to estimate locations and length of motifs, we extract the three most prevalent motifs (positions 138, 0, 82 and length 57, 30, 8) as shown in Fig 15. As already suspected from the instance-based explanations, the motifs contain strong Guanine components. Surprisingly, Guanine seems to dominate all three motifs with no or only little influence of other nucleotide bases. To test whether or not solutions are degenerate, we rank the test sequences according to the inferred *n* ∈ {1, 2, 3} highest scoring motifs (green bars in Fig 15). Interestingly, two motifs are enough to surpass the accuracy of the baseline method (iEnhancer-2L, red dashed line). We therefore conclude that poly(G) sequences are the key for understanding enhancers.

## Applications and limitations

Our experimental section shows very promising results, hence a natural question that arises is: What are promising further applications, even beyond sequence analysis and computational biology, and what are the limitations of ML2Motif? The answer must be split into two parts since ML2Motif itself consists of two distinct parts: gPOIM and convex motifPOIM, both need to be discussed separately in this context.

Convex motifPOIMs are tools to extract driving motifs by mimicking a classifier. Even though the approach is able to find complex, overlapping, and long motifs, some restrictions apply. More specific, the current state of motifPOIM assumes that motifs are localized (they neither change shape nor position) and consists of a finite alphabet (’ACGT’ in our examples). Hence, examples must have same dimensionality (=same length) and to go beyond those restrictions requires further significant research efforts. The same limitations apply to its non-convex predecessor. However, there are plenty of applications where these assumptions are met, e.g. identification of recombination spots [28].

Our proposed feature importance measure gPOIM, on the other hand, is designed to be applicable to any machine learning method and feature representation (e.g. Pse-in-One [29], repDNA [30]). Like POIMs, it takes feature correlations into account but uses a simple sampling based strategy to assess the importances of any feature of interest. Unlike (convex) motifPOIMs, gPOIM is less restricted by specific learning settings. It can be applied to continuous features as well as categorical ones, sequences as well as other structures, e.g. images, trees, etc. Generally, it is not restricted by a specific form of application and/or learning machine. Hence, it could be easily applied to other types of applications such as explanation of most expressive electrode-combination in hand movement recognition with EMG signals [31], change point/anomaly detections in time series for fault detections in wind turbines [32, 33], explanation of important pixel patches in computer vision [6], quantum chemistry [34], and extraction of latent brain states [35]. However, there are two main shortcomings: first, it does not take any non-linear correlations of features into account and second, the number of samples depends on the complexity of the problem.

## Conclusion and outlook

In this work, we have contributed to opening the black box of non-linear learning machines. Our proposed approach, ML2Motif, consists of two techniques: gPOIMs and convex motifPOIMs. ML2Motif nicely extends the DNA motif finding approach SVM2Motif [10], to cope with arbitrary learning machines and feature representations.

gPOIM is a novel algorithmic tool which profoundly improves flexibility and expressiveness of the POIM family. Furthermore, we could derive a convex formulation of the motifPOIM problem that leads to more reliable solutions when compared against its non-convex predecessor. Experiments on artificially generated sequence data as well as on two real-world computational biology datasets demonstrate the benefits of our approach.

Future research will apply gPOIM beyond sequence data. New sampling techniques for faster convergence will be investigated as well as reverse engineering of learning machines aiming to further the understanding gained by the induced motifs. For practical purposes, a Python framework is available at https://github.com/mcvidomi/ML2Motif.

## Supporting information

S1 Appendix Derivations. Further details for extracting motifs by mimicking POIMs and the extension of Theorem 1 and 2 to multiple motifs.
(PDF)
