# Supplementary material for: ML2Motif—Reliable extraction of discriminative sequence motifs from learning machines
Source: PLoS One. 2017 Mar 27;12(3):e0174392. doi: 10.1371/journal.pone.0174392 (PMC5367830; doi:10.1371/journal.pone.0174392)
Supplement: S1 Appendix — Further details for extracting motifs by mimicking POIMs and the extension of Theorem 1 and 2 to multiple motifs. (PDF) [file pone.0174392.s001.pdf]

S1 Appendix

S1.1 Further details for extracting motifs by mimicking POIMs

Definition S.1 (SubPPMs). *A PPM of length k is modeled as a set of D SubPPMs, $D := k - \tilde{k} + 1$ with length $\tilde{k} \leq k$, where SubPPMs are defined by*

$$\tilde{m}_d(m_k, \tilde{k}) := (\tilde{r}, \tilde{\mu}, \sigma),\ \forall\, d = 0, \ldots, D-1\,.$$

Here, $\tilde{\mu} := \mu + d$ and $\tilde{r} := r[d, d+\tilde{k}-1]$, where $r[d, d+\tilde{k}-1]$ is the d-th until the $(d+\tilde{k}-1)$-th column of the PPMs PWM r.

Notation S.1. *Let $\tilde{k} \in \mathbb{N}$ be the value defining the SubPPMs of Def. S.1 and $\mathcal{K} \subset \mathbb{N}, |\mathcal{K}| < \infty$ defining the set of motif lengths, so that $\forall k' \in \mathbb{N}$ with $k' < \tilde{k} : k' \notin \mathcal{K}$ and $T \in \mathbb{N}_0^{\max(\mathcal{K})}$ be the vector defining the number of motifs of any length in $\mathcal{K}$.*

Given Def. S.1 and Notation S.1, the objective function is as follows:

$$f(\eta) = \frac{1}{2} \sum_{k\in\mathcal{K}} \sum_{y\in\Sigma^{\tilde{k}}} \sum_{j=1}^{L} \left(\sum_{t=1}^{T_k} \lambda_{k,t} \sum_{d=0}^{D-1} R_{y,j}(\tilde{m}_d(m_{k,t}, \tilde{k})) - Q_{\tilde{k},y,j} \right)^2, \tag{S.1}$$

where λ indicates the motif relevance and $\eta = (m_{k,t}, \lambda_{k,t}, \tilde{k})_{t=1,\ldots,T_k, k\in\mathcal{K}}$. The associated constrained non-linear optimization problem is thus as follows:

$$\begin{aligned} \min_{(m_{k,t},\lambda_{k,t})_{t=1,\ldots,T_k,k\in\mathcal{K}}} \quad & f(\eta) \\ \text{s.t.} \quad & \epsilon \leq \sigma_{k,t} \leq k, \qquad t = 1,\ldots,T_k\,,\ k\in\mathcal{K} \\ & 1 \leq \mu_{k,t} \leq L-k+1, \quad t = 1,\ldots,T_k\,,\ k\in\mathcal{K} \\ & 0 \leq \lambda_{k,t} \leq \infty, \qquad t = 1,\ldots,T_k\,,\ k\in\mathcal{K} \\ & \epsilon \leq r_{k,t,o,s} \leq 1, \qquad t = 1,\ldots,T_k\,,\ k\in\mathcal{K} \\ & o = 1,\ldots,|\Sigma|, s = 1,\ldots,k\,, \sum_{o=1}^{|\Sigma|} r_{k,t,o,s} = 1\,. \end{aligned} \tag{S.2}$$

S1.2 Extension of Theorem ?? and ?? to Multiple Motifs

Theorem S.1. *Given Notation S.1, suppose that the objective function f of the following optimization problem*

$$\begin{aligned} \min_{r} \quad & f((m_{k,t})_{t=1,\ldots,T_k,k\in\mathcal{K}}) = \frac{1}{2} \sum_{k\in\mathcal{K}} \sum_{y\in\Sigma^{\tilde{k}}} \sum_{j=1}^{L-\tilde{k}+1} \left(\sum_{t=1}^{T_k} \Big(R_{y,j}(m_{k,t}) - S_{\tilde{k},y,j} + c \Big) \right)^2 \\ s.t. \quad & 0 \leq r_{k,t,o,s} \leq 1 \qquad t = 1,\ldots,T_k,\ k\in\mathcal{K}, o = 1,\ldots,4, s = 1,\ldots,k\,, \\ & \sum_{o} r_{k,t,o,s} = 1 \quad t = 1,\ldots,T_k,\ k\in\mathcal{K}, s = 1,\ldots,k\,, \end{aligned}$$

is convex and let r_c^* be the optimal solution, then $\forall c' \in \mathbb{R}\ r_{c'}^* = r_c^*$.

Proof. Let r_c^* be the optimal solution of the objective function f (S.3) with the inequality constraints $h_{k,t,o,s,1} = -r_{k,t,o,s}$ and $h_{k,t,o,s,2} = r_{k,t,o,s} - 1,\ k\in\mathcal{K}, t = 1,\ldots,T_k, o = 1,\ldots,4, s = 1,\ldots,k, i = 1,2$ and the equality constraints $g_k, t, s = \sum_o r_{o,s} - 1,\ k\in\mathcal{K}, t = 1,\ldots,T_k, s = 1,\ldots,k$, and let η and ξ be the Lagrangian multipliers, then the Lagrangian function is as follows

$$\mathcal{L}(r,\eta,\xi) = f(r_c^*;\mu) + \sum_{k\in\mathcal{K}} \sum_{t=1}^{T_k} \sum_{o=1}^{4} \sum_{s=1}^{k} \sum_{i=1}^{2} \eta_{k,t,o,s,i} h_{k,t,o,s,i} + \sum_{k\in\mathcal{K}} \sum_{t=1}^{T_k} \sum_{s=1}^{k} \xi_{k,t,s} g_{k,t,s}\,.$$

The Karush-Kuhn-Tucker(KKT) conditions are satisfied for r_c^*: The primal feasibility conditions ($g_{k,t,s} = 0,\ \mathcal{K}, t = 1, \ldots, T_k, s = 1, \ldots, k$ and $h_{k,t,o,s,i} \leq 0,\ \mathcal{K}, t = 1, \ldots, T_k, o = 1, \ldots, 4, s = 1, \ldots, k, i = 1, 2$) are trivially fulfilled, since r_c^* is a stochastic matrix. Together with the dual feasibility conditions ($\eta \geq 0$) the complementary slackness condition ($\eta_{k,t,o,s,i} h_{k,t,o,s,i} = 0,\ \mathcal{K}, t = 1, \ldots, T_k, o = 1, \ldots, 4, s = 1, \ldots, k, i = 1, 2$) are trivially fulfilled as well, which leaves us to show that the stationarity condition

$$\nabla f(r_c^*; \mu) + \sum_{k \in \mathcal{K}} \sum_{t=1}^{T_k} \sum_{i=1}^{2} \sum_{o} \sum_{s=1}^{k} \eta_{k,t,o,s,1} \nabla h_{k,t,o,s,i} + \sum_{k \in \mathcal{K}} \sum_{t=1}^{T_k} \sum_{s}^{k} \xi_{k,t,s} \nabla g_{k,t,s} = 0$$

is satisfied. Therefore we insert the derivations and reorganize for the Lagrange multipliers ξ , which leads to

$$\begin{aligned} \xi_{k,t,s} \quad = \quad & -\sum_{k \in \mathcal{K}} \sum_{y} \sum_{j} 1_{\{i \in \mathcal{U}(\mu)\}} \Bigg(\sum_{t=1}^{T_k} \prod_{l=1}^{\tilde{k}} r^*_{c,k,t,y_l,j+l} \prod_{\substack{l=1 \\ l \neq t}}^{\tilde{k}} r^*_{c,k,t,y_l,j+l} \\ & -(S_{\tilde{k},y,j+\mu} - c) \prod_{\substack{l=1 \\ l \neq t}}^{\tilde{k}} r^*_{c,k,t,y_l,j+l} \Bigg) + \sum_{k \in \mathcal{K}} \sum_{t=1}^{T_k} \sum_{i=1}^{2} \eta_{k,t,o,s,i}. \end{aligned}$$

With $\xi \in \mathbb{R}$ it holds, that for any $c' \in \mathbb{R}$ $r_{c'}^* = r_c^*$. The fact that f is convex, h is convex and g is affine denotes the KKT conditions as sufficient and concludes the proof. □

Theorem S.2 (Convexity for multiple motifs)**.** *Given Notation 1, let D be a convex set, $m_k \in D$ a probabilistic motif, S a gPOIM, such that $S_{\tilde{k},y,j} \in \mathbb{R}$ for $y \in \Sigma^{\tilde{k}}$ and $j = 1, \ldots, L - \tilde{k} + 1$, $\mu \in [1, L - k + 1]$, $c \in \mathbb{R}$ and $S_{\lfloor}$ the element wise minimum of S then, if $c \geq \mathbb{1}_{\{S_{\lfloor} < 0\}} S_{\lfloor} + \mathbb{1}_{\{S_{\lfloor} < T_k\}} T_k$ it holds that*

$$f((m_{k,t})_{t=1,\ldots,T_k, k \in \mathcal{K}}) \quad = \quad \frac{1}{2} \sum_{k \in \mathcal{K}} \sum_{y \in \Sigma^{\tilde{k}}} \sum_{j=1}^{L-\tilde{k}+1} \left(\sum_{t=1}^{T_k} R_{y,j}(m_{k,t}) - (S_{\tilde{k},y,j} + c) \right)^2$$

is convex.

Proof. We have to proof the following inequality to show convexity of $f(m_k)$

$$\begin{aligned} || \sum_{t=1}^{T_k} R(\Phi r_{k,t} + (1 - \Phi) s_{k,t}; \mu) - (S + c') ||_2^2 \leq \Phi || \sum_{t=1}^{T_k} R(r_{k,t}; \mu) - (S + c') ||_2^2 \\ + (1 - \Phi) || \sum_{t=1}^{T_k} R(s_{k,t}; \mu) - (S + c') ||_2^2 \end{aligned}$$

which is, for the case $j \notin \mathbb{1}_{\{i \in \mathcal{U}(\mu)\}}$, trivially fulfilled for $c' \in \mathbb{R}$. This, due to the fact, that a sum of convex functions is convex, leaves us with showing the following inequality

$$\begin{aligned} \big(\sum_{t=1}^{T_k} \Phi a_t + (1 - \Phi) b_t - (S_{\tilde{k},y,j} + c') \big)^2 \leq \Phi \big(\sum_{t=1}^{T_k} a_t - (S_{\tilde{k},y,j} + c') \big)^2 \\ + (1 - \Phi) \big(\sum_{t=1}^{T_k} b_t - (S_{\tilde{k},y,j} + c') \big)^2 , \end{aligned} \qquad \text{(S.3)}$$

where we replaced the PWM products $\prod_{l=j}^{k+j} r_{k,t,y_l,l}$ and $\prod_{l=j}^{k+j} s_{k,t,y_l,l}$ by a_t and b_t for more transparency. After resolving and transforming Eq. (S.3) shortens to

$$\Phi^2 \sum_{t=1}^{T_k} a_t^2 + 2\Phi \sum_{t=1}^{T_k} a_t b_t - 2\Phi^2 \sum_{t=1}^{T_k} a_t b_t \leq \Phi \sum_{t=1}^{T_k} a_t^2 + 2\Phi(S_{\tilde{k},y,j} + c')^2. \tag{S.4}$$

Since $-2\Phi^2 \sum_{t=1}^{T_k} a_t b_t \leq 0$ and $\Phi^2 \sum_{t=1}^{T_k} a_t^2 \leq \Phi \sum_{t=1}^{T_k} a_t^2$, Eq. (S.4) reduces to $\sum_{t=1}^{T_k} a_t b_t \leq (S_{\tilde{k},y,j} + c')^2$. The fact that the maximum of $\sum_{t=1}^{T_k} a_t b_t$ is T_k, concludes the proof for $c \geq c'$ with $c' = \mathbb{1}_{\{\min(S)<0\}} S_{\lfloor} + \mathbb{1}_{\{S_{\lfloor} < T_k\}} T_k$. □
